# Neurophysiology of threat processing bias in combat‐related post‐traumatic stress disorder

**DOI:** 10.1002/hbm.24800

**Published:** 2019-10-04

**Authors:** Bambi L. DeLaRosa, Jeffrey S. Spence, Nyaz Didehbani, Gail D. Tillman, Michael A. Motes, Christina Bass, Michael A. Kraut, John Hart

**Affiliations:** ^1^ School of Behavioral and Brain Sciences The University of Texas at Dallas Dallas Texas; ^2^ Department of Radiology The Johns Hopkins University School of Medicine Baltimore Maryland; ^3^ Department of Neurology and Neurotherapeutics The University of Texas Southwestern, Medical Center Dallas Texas

**Keywords:** electroencephalography, post‐traumatic stress disorder, theta, threat processing, veterans

## Abstract

Post‐traumatic stress disorder (PTSD) is a debilitating condition that may develop after experiencing a traumatic event. Combat exposure increases an individual's chance of developing PTSD, making veterans especially susceptible to the disorder. PTSD is characterized by dysregulated emotional networks, memory deficits, and a hyperattentive response to perceived threatening stimuli. Recently, there have been a number of imaging studies that show structural and functional abnormalities associated with PTSD; however, there have been few studies utilizing electroencephalography (EEG). The goal of this study was to characterize **EEG brain dynamics in individuals with PTSD, in order to better understand the neurophysiological underpinnings of some of the salient features of PTSD, such as threat‐processing bias. Veterans of Operation Enduring Freedom/Iraqi Freedom completed an implicit visual threat semantic memory recognition task with stimuli that varied on both category (animals, items, nature, and people) and feature (threatening and nonthreatening) membership, including trauma‐related stimuli. Combat veterans with PTSD had slower reaction times for the threatening stimuli relative to the combat veterans without PTSD (VETC). There were trauma‐specific effects in frontal regions, with theta band EEG power reductions for the threatening combat scenes in the PTSD patients compared to the VETC group. Additionally, a moderate negative correlation was observed between trauma‐specific frontal theta power and hyperarousal symptoms as measured by clinically administered PTSD scale. These findings complement and extend current models of cortico‐limbic dysfunction in PTSD. The moderate negative correlation between frontal theta power and hyperarousal endorsements suggests the utility of these measures as therapeutic markers of symptomatology in PTSD patients.

## 1 INTRODUCTION

Post‐traumatic stress disorder (PTSD) is a severely debilitating disorder that may develop after an individual experiences a traumatic event, resulting in a dysfunctional emotional state with a myriad of deleterious cognitive sequelae. PTSD is characterized by dysregulated emotional networks, memory deficits, and a heightened response to perceived threatening stimuli. PTSD is distinguishable from other anxiety related disorders by memory disruptions and dysfunctional threat processing (Desmedt, Marighetto, & Piazza, 2015; Zoellner, Pruitt, Farach, & Jun, 2014).

The disorder is difficult to treat, and current treatment options are not effective for all individuals. Approximately, 7.7 million Americans suffer from PTSD, with veterans being especially affected (Kessler, Chiu, Demler, & Walters, 2005; Kok, Herrell, Thomas, & Hoge, 2012). Veterans of operation Iraqi freedom (OIF) and operation enduring freedom (OEF) have high incidences of PTSD, with prevalence strongly related to exposure to threatening combat environments (Kok et al., 2012; Renshaw, 2011; Seal et al., 2009; Seal, Bertenthal, Miner, Sen, & Marmar, 2007).

There is accumulating evidence that memory retrieval deficits in PTSD result from hyper‐responsive limbic systems and a failure of top–down cortical inhibition (Pitman et al., 2012). In addition, there is good evidence that memory retrieval mechanisms in individuals with PTSD are biased toward threatening, and specifically trauma‐related, stimuli (Bar‐Haim, Lamy, Pergamin, Bakermans‐Kranenburg, & Van Ijzendoorn, 2007; Cisler et al., 2011; Pergamin‐Hight, Naim, Bakermans‐Kranenburg, van IJzendoorn, & Bar‐Haim, 2015). This threat processing bias has been characterized behaviorally; however, relatively little is known about the neurophysiological underpinnings of this threat processing bias found in PTSD.

A threat‐processing bias may be defined as a differential processing of threatening, relative to nonthreatening, stimuli and may be manifested as delayed reaction time to stimulus, changes in event‐related potential amplitudes, or changes in electroencephalography (EEG) oscillatory power. Due to its millisecond temporal resolution, EEG is ideally suited to investigate the neurophysiological origins of memory deficits and valence (threatening vs. nonthreatening) biases found in PTSD. Characterizing EEG oscillatory dynamics during memory retrieval, including retrieval of threatening stimuli, can help clarify the bases of cognitive dysfunctions in PTSD, and thus guide clinical treatment. Cortical theta oscillations are of particular interest because of their potential involvement in threat processing. Mueller, Panitz, Hermann, and Pizzagalli (2014) investigated source‐localized EEG oscillations in healthy humans, and found that fear expression is associated with anterior mid‐cingulate theta activity. In addition, our group previously found differential theta power changes within the frontal cortices for threatening objects during a simple visual discrimination task. The task assessed visual object retrieval as it is influenced by category (e.g., animals, items, nature, and people) and valence (e.g., threatening, nonthreatening) membership. There was greater theta power for threatening than nonthreatening images observed in frontal electrodes (DeLaRosa et al., 2014).

There is established evidence for a threat‐related bias in anxiety disorders, including PTSD, although the mechanisms and neurophysiology underlying this bias remains unknown. Awaiting clarification is whether this hypersensitivity to threatening stimuli is driven by a hyperactive automatic threat detection network (Beck & Clark, 1997; Monk et al., 2008; van den Heuvel et al., 2005; Williams, Watts, MacLeod, & Mathews, 1988), or failure of prefrontal cortical regulation of attention (Bishop, Duncan, Brett, & Lawrence, 2004; Eysenck, Derakshan, Santos, & Calvo, 2007; Wu et al., 2010). Helping to disambiguate these mechanisms are studies utilizing threatening stimuli that vary as to their category membership (trauma‐specific or a general threat). For example, a hyperactive and automatic threat detection mechanism, primarily involving the amygdala, may be less affected by content specificity due to the automatic nature of threat classification; whereas, cortical involvement may be more sensitive to category membership (Bishop et al., 2004; LeDoux, 1998).

In this study, we utilized a visual discrimination task that implicitly probed semantic memory object recognition of stimuli that varied based on both categorical and featural components. The categories were animals, items, nature, and people, and the feature of valence was either threatening or nonthreatening. Trauma‐specific stimuli comprised weapons and combat‐theater‐specific scenes. This task allowed for the investigation of threat‐processing bias as it relates to both trauma‐specific threats and other general threatening stimuli.

We investigated both reaction time and cortical theta power changes of veterans with combat‐related PTSD (PTSD group) compared to combat‐exposed veterans without PTSD (VETC group) in response to a visual discrimination task that implicitly probed threat‐processing. We hypothesized that the PTSD group would have delayed reaction times for the threatening stimuli relative to the VETC group, suggesting interference of threat processing on cognitive function for the PTSD group (Bar‐Haim et al., 2007; Carretié, Hinojosa, Martín‐Loeches, Mercado, & Tapia, 2004; Zoellner et al., 2014). To probe whether this threat bias was generalized for all threats or specific to threatening trauma‐related stimuli, we assessed category by feature by group interactions (Cisler et al., 2011; Pergamin‐Hight et al., 2015). Electrophysiological predictions were that frontal theta would be greater for threatening than for nonthreatening stimuli at frontal recording sites for both groups, and that, relative to the VETC group, the PTSD group would have reduced frontal cortical theta indicating impaired fronto‐limbic modulation (Calley et al., 2013; Cavanagh & Shackman, 2015; DeLaRosa et al., 2014; Mueller et al., 2014; Pitman et al., 2012). We also assessed group by category by valence interactions in order to evaluate the contribution of trauma‐specific stimuli. To relate these findings to PTSD symptomatology, frontal theta‐band EEG power changes in response to threatening trauma‐related stimuli were correlated with the Clinically‐Administered PTSD Scale (Association, 2003; Blake et al., 1990). We predicted that frontal theta would negatively correlate with PTSD hyperarousal symptomatology, that is, that lower frontal theta would correlate with a higher hyperarousal score, suggesting a modulatory role of frontal cortical theta (Bishop et al., 2004; Mueller et al., 2014).

## 2 METHODS AND MATERIALS

### 2.1 Recruitment

Veterans previously deployed to combat regions from 2001 to present (e.g., OEF, OIF, and operation new dawn [OND]) were recruited from the community. Recruitment focused on military installations, veteran affairs hospitals, veteran centers, local universities and colleges with veteran enrollment, and various nonprofit veteran‐associated service organizations. Interested participants contacted researchers at the University of Texas at Dallas Center for BrainHealth (CBH) for an initial phone screening to determine eligibility and to discuss the study in general. Potential participants who met initial qualifications had a follow‐up meeting at CBH. All participants gave informed, written consent to participate. The experiment was approved by the Institutional Review Boards for the University of Texas at Dallas, the University of Texas Southwestern Medical Center, and by the Army Human Research Protection Office. Exclusion criteria included presence of substance abuse (within the past 3 months), history of psychotic symptoms, Parkinson's disease, multiple sclerosis, obsessive compulsive disorder, brain tumor, stroke, abnormalities or trauma to the skull, evidence of structural brain damage (from structural magnetic resonance imaging scan), current pregnancy, seizure, cardiac abnormalities, or any implanted metal objects near the head. If a participant met criteria, approved medications needed to be stable for 4–6 weeks prior to beginning participation in the study. Following the initial interview, qualifying participants then returned to the CBH for baseline testing.

### 2.2 Baseline testing

Baseline testing consisted of self‐evaluation forms, a clinical assessment by a trained licensed clinician, EEG, and functional and structural imaging. All participants were clinically evaluated by a licensed psychologist. Following an initial assessment, a licensed psychologist reviewed scores from the initial screen which included the Mississippi PTSD scale, PTSD checklist‐military version (PCL‐M), and quick inventory of depressive symptomology self‐report to confirm inclusion to either the PTSD group or the control group. Any control participant that demonstrated elevated scores or signs and symptoms of PTSD, were fully evaluated with the clinician administered PTSD scale.

Clinician‐administered evaluations included the clinically administered PTSD scale (CAPS) for diagnostic and statistical manual of mental health disorders (Blake et al., 1990) and the structured clinical interview for DSM‐IV‐TR‐Axis I disorders‐patient version (First, 1995). The CAPS measures symptom severity and includes 30 interview questions focused on DSM‐IV‐TR criteria for PTSD (Association, 2003). This includes symptoms of re‐experiencing, avoidance and numbing, hyperarousal, subjective distress, onset, duration, symptom severity, and functional impairment.

### 2.3 Participants

Those combat‐exposed veterans who met inclusion criteria, and were identified as not having PTSD, after clinical assessment, served as control participants. There were a total of 44 participants tested, with 29 in the PTSD group and 15 in the control group. Two participants in the control group were excluded, one due to poor quality and thus unusable EEG data, and one who failed to meet inclusion criteria after clinical assessment. Ultimately, we analyzed data from 42 participants (nPTSD = 29, nVETC = 13). There were no significant differences of age and years of active duty between the two groups. Summary of demographics and clinical measures are detailed in Table 1.

**Table 1:** Demographics and clinical measures for the veterans with PTSD group (PTSD) and the veterans without PTSD group (VETC)

Group	PTSD (*n* = 29)		VETC (*n* = 13)	
*Demographics*	Mean (*SD*)	Range	Mean (*SD*)	Range
Age	32.24 (7.38)	24–51	32.00 (5.70)	26–48
Education in years	14.14 (2.17)	12–19	16.15 (1.21)	14–18
Years of active duty	6.405 (4.41)	1.5–20.52	4.867 (2.93)	1.0–12.5
Gender		27/2 (M/F)		10/3 (M/F)

Race	17/4/8 (Caucasian/Hispanic/other)		10/2/1 (Caucasian/Hispanic/other)	
*Clinical* a	Mean (*SD*)	Range	Mean (*SD*)	Range
Inventory of psychosocial functioning	3.09 (0.76)	1.52–4.920	2.50 (0.69)	1.39–3.67
Clinician‐administered PTSD scale	70.10 (23.36)	22.00–109.00	NA	NA
Quick inventory of depression scale	11.14 (4.96)	3.00–20.00	4.39 (4.99)	0.00–16.00
Mississippi scale for combat related PTSD	103.90 (18.56)	50–131	60.50 (12.81)	39.00–91.00

Abbreviations: PTSD, post‐traumatic stress disorder; VETC, veterans without PTSD.

a There were significant group differences for the listed clinical measures including, Inventory of Psychosocial Functioning [*t*(22) = 2.39, *p* = .03], Quick Inventory of Depression Scale [*t*(23) = 4.06, *p* < .001], and the Mississippi Scale for Combat Related PTSD [*t*(29) = 8.59, *p* < .001].

### 2.4 Stimuli

**Figure 1:**
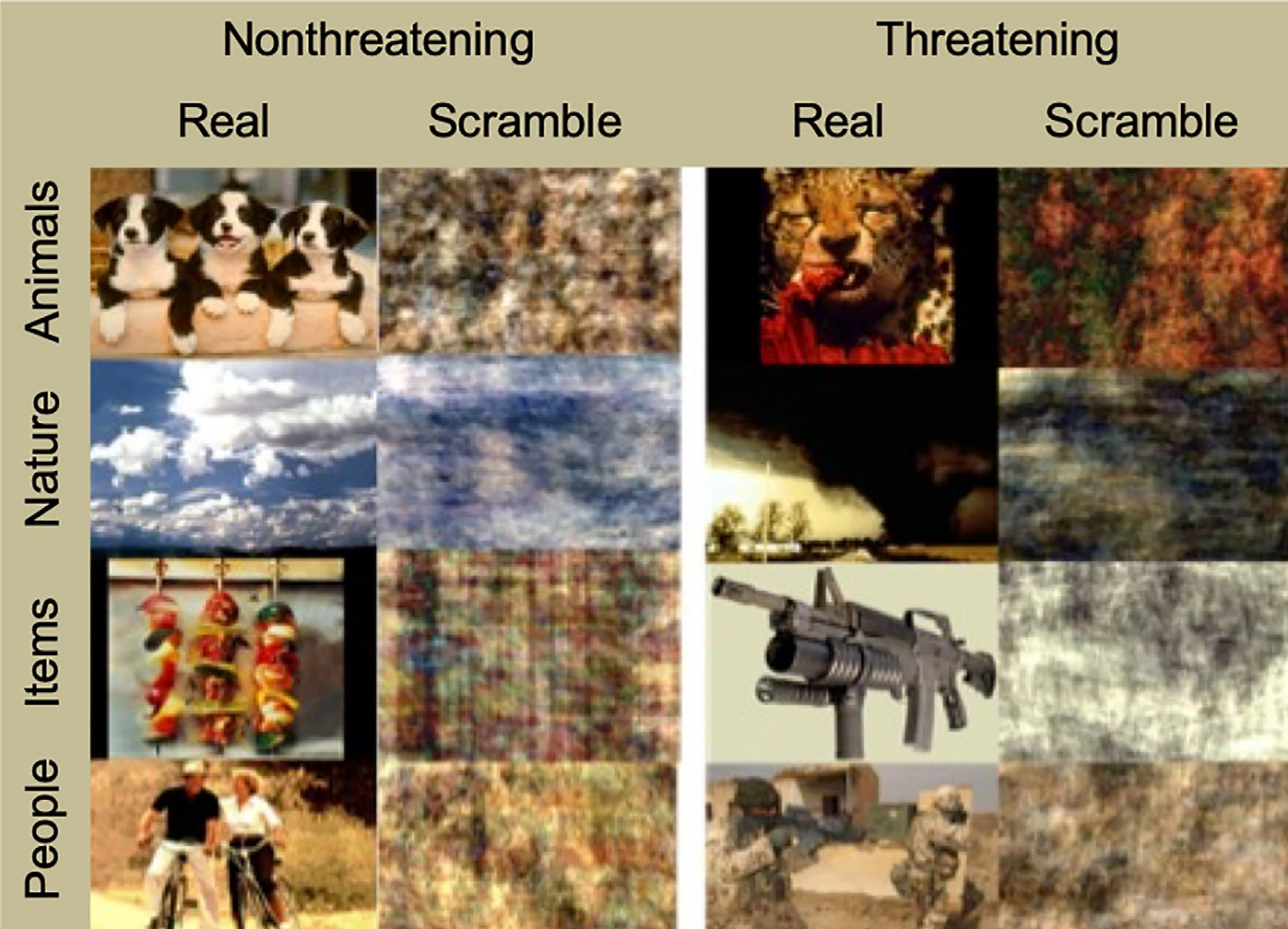
Example of stimuli and visual depiction of categorical grouping. Stimuli were derived from four categories: animals, nature scenes, handheld/manipulable items (weapons and food), and people in scenes (military personnel in OIF/OEF/OND combat scenes and civilians in pleasant scenes). In addition, the stimuli included both threatening and nonthreatening examples in each category giving a total of eight groupings of images (16 images in each group). OIF, operation Iraqi freedom; OEF, operation enduring freedom; OND, operation new dawn

One hundred twenty‐eight colored images depicting various objects and environmental scenes were used in the present study. The images consisted of 96 colored images from the international affective picture set (Lang, Bradley, & Cuthbert, 2008) of normed pictures plus an additional 32 stock images selected for the categories of combat scenes and weapons specific to OEF/OIF (Calley et al., 2013; DeLaRosa et al., 2014). The visual complexity and resolution of the additional images were comparable to those of the IAPS images. Each image was modified to create a matched, scrambled version by randomizing and recombining the phase information of the original picture (Haxby, Hoffman, & Gobbini, 2002; Hoffman & Haxby, 2000). Thus, there were 256 total pictures, 128 pictures of “real” stimuli and 128 scrambled versions of those pictures (Figure 1).

Stimuli were derived from four categories: animals, handheld/manipulable items (weapons and food), nature scenes, and people in scenes (military personnel in OIF/OEF/OND combat scenes and civilians in pleasant scenes). The stimuli were from the two ends on the IAPS pleasantness scale. In addition, the categories of stimuli were also split between threatening and nonthreatening, based on normed ratings (Lang et al., 2008).

### 2.5 Behavioral procedures

The 256 pictures of real and nonreal “scrambled” items were pseudorandomized and presented individually using a Stim^2^ system (Compumedics Neuroscan). The stimuli were presented on a computer screen approximately 1 m in front of the subject. Each stimulus was presented for 2,700 ms, with a pseudorandom jittered interstimulus interval average of 2,300 ms. Subjects were instructed to push a button under their right index finger to indicate that they perceived an item to be real (an item they recognized), and to push a button under their right middle finger for a nonreal item (a scrambled image). Total task time was 20 min.

### 2.6 EEG recording

Continuous EEG was recorded from a 64‐electrode Neuroscan Quickcap using Neuroscan SynAmps2 amplifiers and Scan 4.3.2 software, with a reference electrode located near the calvarial vertex. Data were sampled at 1 kHz with impedances typically below 10 kΩ. Additionally, bipolar electrooculographic data were recorded from two electrodes to monitor blinks and eye movements (positioned vertically at the supraorbital ridge and lower outer canthus of the left eye). The continuous EEG data were offline high‐pass filtered at 0.5 Hz and low‐pass filtered at 30 Hz using a finite impulse response filter.

### 2.7 EEG preprocessing

We analyzed the EEG data using scripts developed in our lab that implement functions from EEGLAB version 13.1 (Delorme & Makeig, 2004) running under MATLAB 7.11.0. Preprocessing consisted of down‐sampling to 512 Hz, removing data recorded from poorly functioning electrodes, and correcting for stereotyped artifacts including eye blinks, lateral eye movements, muscle, line noise, and heart rate using the “Runica” algorithm (Delorme & Makeig, 2004; Jung et al., 2000), an implementation of the logistic infomax independent component analysis algorithm of Bell and Sejnowski (1995). Stereotyped artifacts were identified by visual inspection of the spatial and temporal representation of the independent components. Continuous data were then segmented into two‐second nonoverlapping epochs spanning from 500 ms before to 1,500 ms after the presentation of the visual stimuli. Epochs containing high amplitude, high frequency muscle noise, and other irregular artifacts were removed retaining on average 75% of all epochs. Finally, missing electrodes were interpolated and data were re‐referenced to the average reference (Junghöfer, Elbert, Tucker, & Rockstroh, 2000).

### 2.8 Event‐related time‐frequency analysis

Event‐related spectral perturbations (Makeig, 1993) were calculated using the “newtimef” function of EEGLAB toolbox. Thirty linearly spaced frequencies from 1 to 30 Hz were estimated using Hanning Fast Fourier Transform tapering in 50 time windows (−384 to 895 ms). We performed single‐trial baseline correction using the prestimulus 500 ms interval as baseline (Grandchamp & Delorme, 2011).

### 2.9 Statistical analysis

Reaction times (ms) were obtained for every subject and for each trial, and trials were rejected if their reaction times were greater than the 99.5th percentile of a fitted gamma function to each subject's reaction time distribution. A gamma function was used because the reaction time data was right‐skewed, rendering standard deviation methods of outlier detection less applicable. Gamma fitting was implemented in MATLAB using the gamfit function. No more than two trials were discarded per subject per condition. Reaction time values, using correct trials only, were subsequently log transformed and averaged for each subject.

Electrode FPZ was used to determine frontal time‐frequency effects (Calley et al., 2013; DeLaRosa et al., 2014). Peak theta estimates were extracted per subject by averaging over 4–8 Hz per condition, and peak values were determined by the local maximum within the time window of 100–800 ms poststimulus presentation.

Spectral variables, described above, were modeled as additive effects of group (PTSD, VETC), condition (real, scrambled), valence (nonthreatening, threatening), and category (with animals and nature defining a “nontrauma” subcategory; items and people defining a “trauma” subcategory), in addition to interactions among each combination of these effects. This linear statistical model was implemented in SAS (Cary, NC) using *Proc Mixed* for inference on planned contrasts of parameter estimates. The mixed model also included two random terms to account for subject‐level variability and trial‐level variability within each subject. Behavioral variables were modeled similarly, except that condition effects were not included. Effects of group, valence, and category were tested within the real condition only.

Behavioral contrasts focused on reaction time group differences as influenced by valence effects and category effects (trauma vs. nontrauma) within the real condition. This contrast was followed with group by valence differences. Regarding spectral variables, we first tested whether the PTSD group showed a category (trauma vs. nontrauma) difference, relative to the VETC group, with respect to the effect of valence on theta power, after removing any possible effect of the paired scrambled images on power. Statistically, this test represents a 1‐degree‐of‐freedom test of a four‐factor interaction. We expected this result primarily due to an abnormal response to threatening trauma images (weapons and combat scenes) for the PTSD group. Therefore, we followed the four‐factor interaction test with contrasts that hierarchically narrowed down to the PTSD trauma‐specific effect of threat on theta power. These latter contrasts included the group by category difference (trauma vs. nontrauma) with respect to threatening valence on theta power, after correcting for a possible effect of scrambled images (a conditional three factor interaction); the group differences on the subdivided threatening trauma stimuli (combat scenes), after scramble correction (a conditional two‐factor interaction); and finally, the group differences on the subdivided threatening trauma stimuli (weapons), after scramble correction (a conditional two‐factor interaction). All planned contrasts were directional hypotheses, and we controlled alpha inflation due to the multiple hierarchical tests, following the four‐factor interaction test, by maintaining the false‐discovery rate at 5%.

To assess the relationship between task‐related cortical oscillatory dynamics and symptom expression in the PTSD group, theta power changes for trauma related stimuli were correlated with clinical assessments. The responses to the scrambled images of the trauma‐related threatening combat scenes were used as the baseline to obtain difference values. This was done to isolate the effect of threatening combat scenes and eliminated condition induced changes in power. Differential theta power values at FPZ were correlated with the Clinical‐Administered PTSD Scale for DSM‐IV (Blake et al., 1990).

## 3 RESULTS

### 3.1 Behavioral results

**Figure 2:**
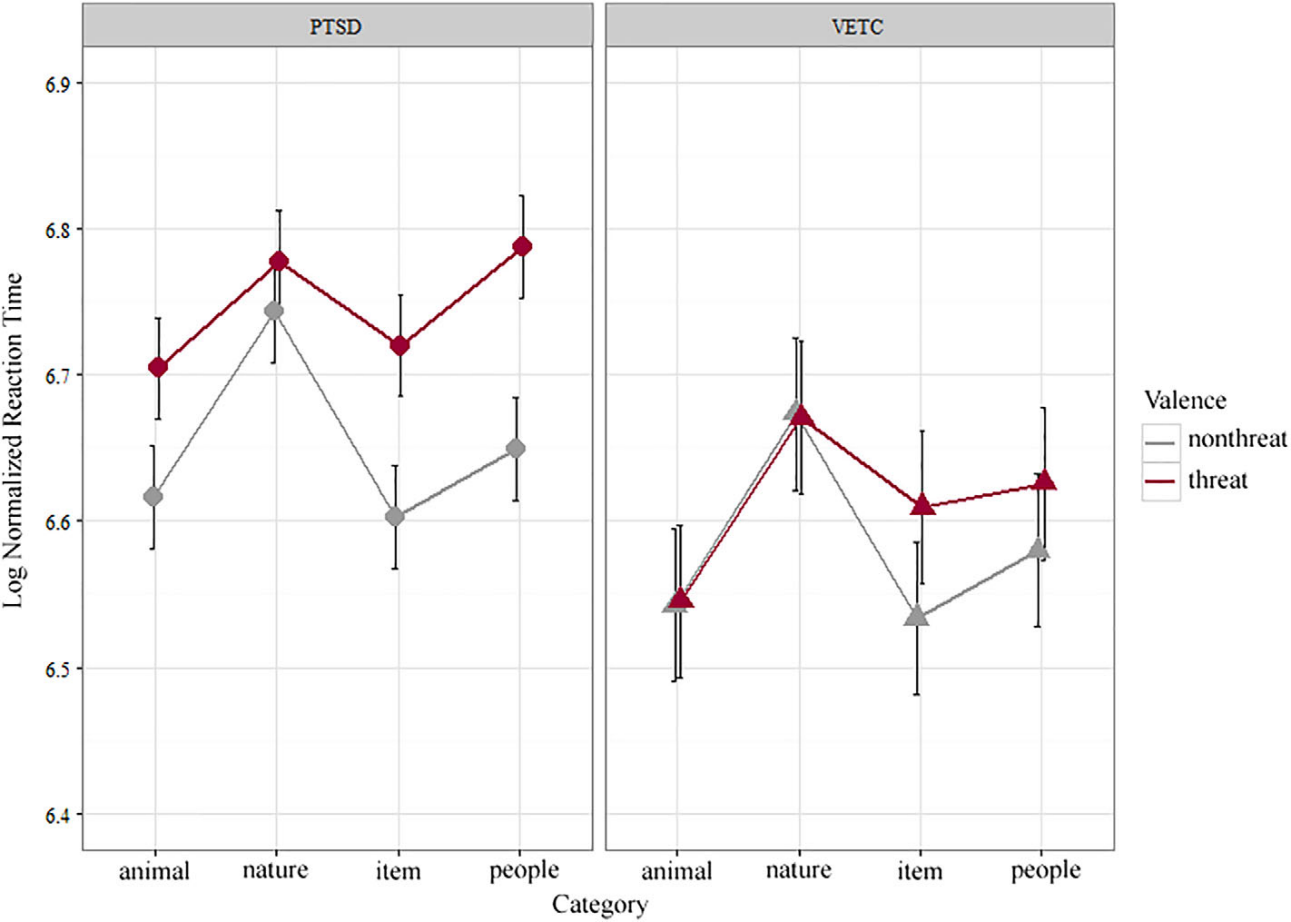
Log normalized reaction time plotted as a function of group by category by valence. *Animal* and *Nature* comprised the nontrauma category and *item* and *people* comprised the trauma category. *People threatening* were combat scenes specific to OEF/OIF, and *threatening items* were weapons specific to OEF/OIF. The PTSD group had slower reaction times for the threatening stimuli relative to the VETC group, and there were no differences for the nonthreatening stimuli, *t*(599) = −2.24, *p* = .01. Average Reaction times for the real images by category in milliseconds are as follows: PTSD threatening (*M* = 873.26 ms, *SEM* = 23.17), PTSD nonthreatening (*M* = 788.32 ms, *SEM* = 23.17), VETC threatening (*M =* 758.70 ms, *SEM* = 34.61), VETC nonthreatening (*M* = 733.43 ms, *SEM* = 34.61). OIF, operation Iraqi freedom; OEF, operation enduring freedom; PTSD, post‐traumatic stress disorder; VETC, veterans without PTSD

Overall accuracy was high with both groups of veteran participants successfully completing the task. There were no group accuracy differences between the PTSD group (*M* = 0.91, *SEM* = 0.02) and the VETC group (*M* = 0.88, *SEM* = 0.02), *F*(1,39) = 0.97, *p* = .33. The three factor interaction contrast within the real threatening images of group by valence by category, collapsing over trauma (items and people) versus nontrauma (animal and nature) showed no differences in reaction time, *t*(599) = 0.10, *p* = .55. However, there were valence dependent reaction time differences between groups, a significant two‐factor interaction *t*(599) = −2.24, *p* = .01 (Figure 2). The PTSD group was slower for the threatening real images (*M* = 6.75, *SEM* = 0.03) relative to the VETC group (*M* = 6.61, *SEM* = 0.04), *t*(50.7) = 2.58, *p* = .006; whereas the reaction times were similar between groups for the nonthreatening real images.

Taken together, these indicate that the longer reaction time to the threatening images for the PTSD group was not driven by the presence of the trauma‐specific images, but instead was a general delay to all threatening images regardless of category membership.

### 3.2 Event‐related EEG power results

#### 3.2.1 FPZ peak theta power

**Figure 3:**
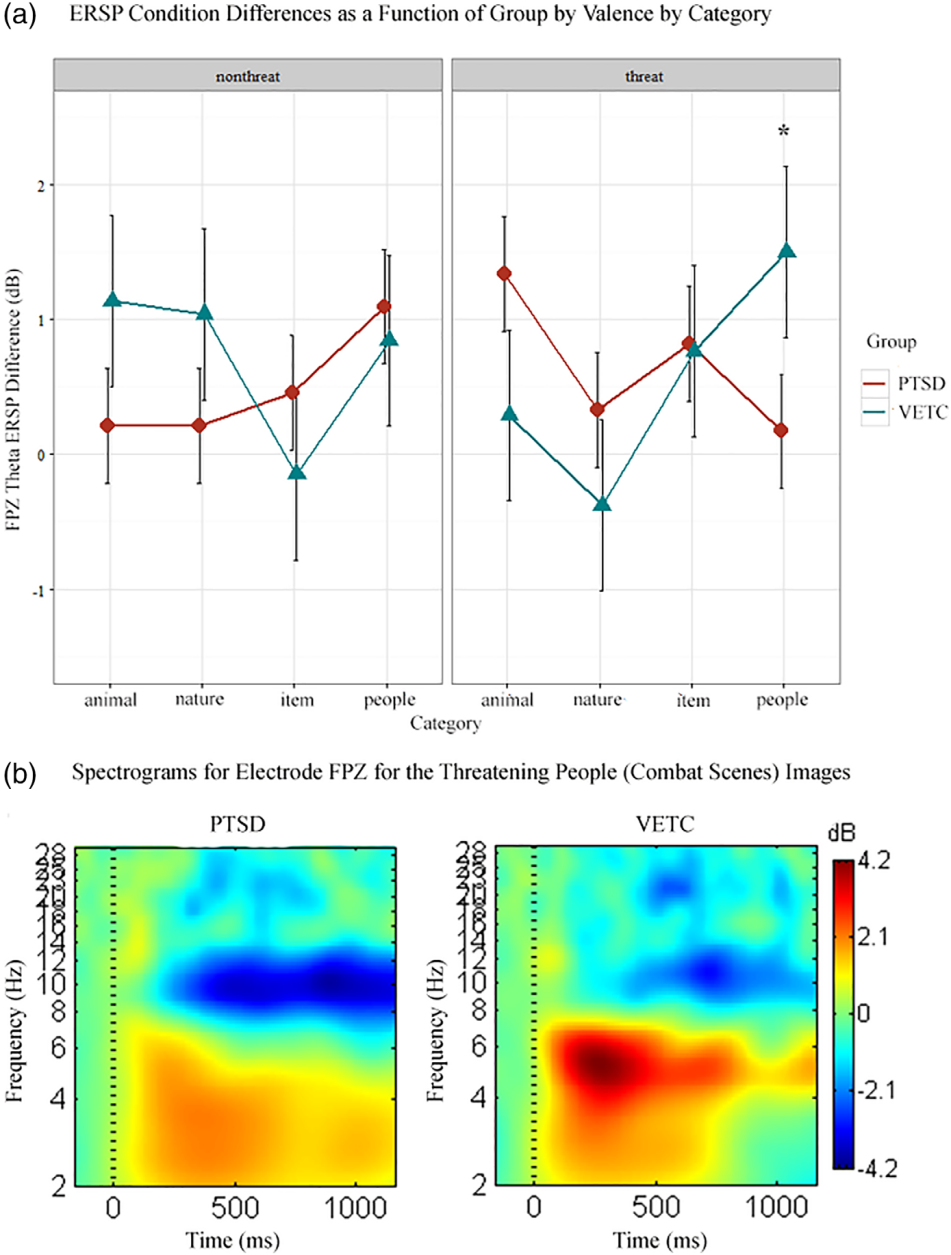
(a) ERSP condition difference plotted as a function of group by valence by category for electrode FPZ. *Animal* and *Nature* comprised the nontrauma category and *item* and *people* comprised the trauma category. *People threatening* were combat scenes specific to OEF/OIF, and *threatening items* were weapons specific to OEF/OIF. The PTSD group had lower conditional theta power for the threatening combat images (people) relative to the VETC group, denoted by asterisk and tested as a single contrast as well as contrasts against the other levels (see Table 2). (b) Spectrograms for electrode FPZ for the threatening people category (combat scenes). The left spectrogram is the PTSD group average, and the right spectrogram is the VETC group average. The PTSD group had lower theta power for the threatening combat images relative to the VETC group. OIF, operation Iraqi freedom; OEF, operation enduring freedom; PTSD, post‐traumatic stress disorder; VETC, veterans without PTSD

We found statistically significant differences in electrode FPZ peak theta power for the four factor interaction contrast group by condition by valence by category, *t*(600) = −2.61, *p* = .004 (Figure 3). The hierarchical follow‐up contrast investigating group by condition by category within the threatening valence was similarly significantly different, *t*(600) = −1.99, *p* = .02. To investigate if this effect was differentially influenced by the two categories of trauma‐specific images (people, items), the following contrasts subdivided the traumatic threatening images into combat scenes (people category) and weapons (item category). There was a statistical difference for the group by condition for the combat scenes, *t*(600) = −1.74, *p* = .04, but there was no difference for the group by condition interaction for the weapons category, *t*(600) = 0.07, *p* = .47. Thus, the planned contrast probing trauma versus nontrauma stimuli was driven by the threatening people (combat scenes) category (see Table 2 for the full list of tests probing the differences between groups on threatening traumatic scenes).

**Table 2:** Details of hierarchical planned contrasts. (P_t_‐C_t_)_threat_ is our primary interest. Thus, it appears as a single contrast and as contrasts against other levels in the higher order interactions

Description	Contrast	*t*	*df*	*p*
Group x condition x category x valence	[(P_t_ ‐ C_t_)_threat_ − (P_n_ ‐ C_n_)_threat_] − [(P_t_ ‐ C_t_)_nonthreat_ − (P_n_ ‐ C_n_)_nonthreat_]	−2.61	600	.004^a^
Group x condition x category (for threatening valence)	[(P_t_ ‐ C_t_)_threat_ − (P_n_ ‐ C_n_)_threat_]	−1.99	600	.02^a^
Group x condition (for trauma threatening weapons)	(P_t_‐C_t_)_threat_	0.07	600	.53
Group x condition (for trauma threatening combat scenes)	(P_t_‐C_t_)_threat_	−1.74	600	.04^a^

*Note*: P, PTSD group; C, VETC group; t, trauma category; n, nontrauma category.

a false‐discovery rate (FDR) = 5%.

#### 3.2.2 Relationship between PTSD symptom severity and frontal theta for threatening trauma‐specific images

**Figure 4:**
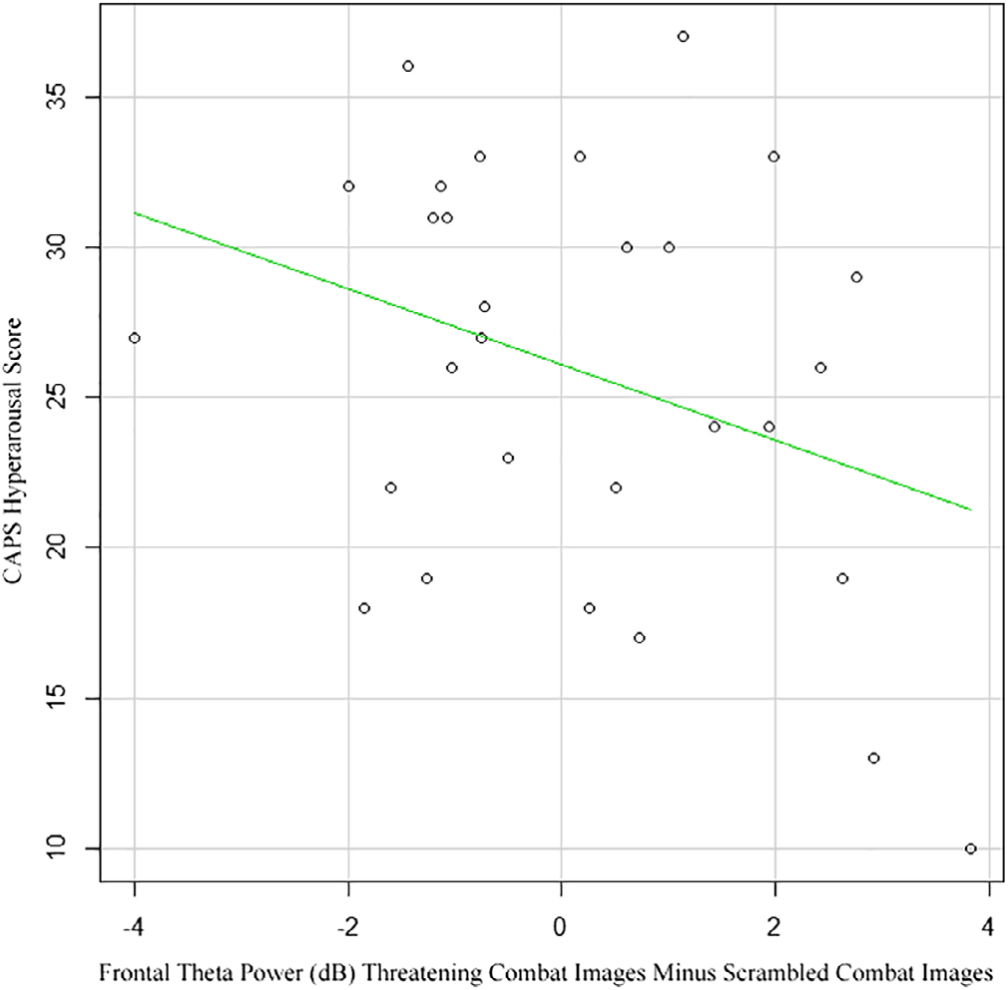
Correlation between differential theta power at electrode FPZ for the threatening combat images with clinically administered PTSD scale (CAPS) hyperarousal symptom score. There was a moderately negative correlation between FPZ theta power and the CAPS hyperarousal score (CAPS, Criterion D), *r* = −.33, *n* = 29, *p* = .04

To investigate the relationship between frontal theta power with clinical symptomatology, a Pearson's correlation analysis was performed within the PTSD group between frontal theta power for the combat images and the CAPS hyperarousal score. There was a moderately negative correlation between FPZ theta power and the CAPS hyperarousal score (CAPS, Criterion D), *r* = −.33, *n* = 29, *p* = 0.04, indicating an association between lower frontal theta for threatening trauma‐specific images and higher hyperarousal scores (Figure 4).

## 4 DISCUSSION

In this study of cortical oscillatory EEG dynamics during a semantic threat memory task, we found that, when compared with combat veterans without PTSD, combat veterans with PTSD had both trauma‐specific and general threat processing disturbances, corroborating current models of cortico‐limbic dysfunction in PTSD. We found behavioral differences, with slower reaction times in the PTSD patients for the threatening stimuli, irrespective of category membership. This suggests a generalized dysfunctional threat response whereby the presence of threatening information impedes cognitive operations in PTSD patients. There were also trauma‐specific effects in frontal regions, with theta band power reductions for the threatening combat scenes in the combat‐related PTSD patients compared to the non‐PTSD controls. These frequency band‐specific effects negatively correlated with hyperarousal symptoms on the CAPS, suggesting the utility of these measures as therapeutic markers of this debilitating symptom in these patients.

### 4.1 Behavioral

The test stimuli consisted of real and scrambled images, with the participants engaged in a choice response task, deciding whether the image was “real” or “not real.” In addition, the images depicted various categories of stimuli (animals, items, nature, and weapons) and were split between a factor of valence (threatening and nonthreatening). This provided an opportunity to implicitly probe semantic object recognition without explicitly asking subjects to categorize or appraise valence membership. Since it was a simple task, accuracy effects were not predicted, and both groups had high accuracy scores, indicating that they were engaged in the task. For reaction time, since threat‐processing interference has previously been reported in the literature (Bar‐Haim et al., 2007; Buckley, Blanchard, & Neill, 2000; Olatunji, Armstrong, McHugo, & Zald, 2013) it was predicted that the veterans with PTSD (PTSD group) would be slower to the threatening stimuli compared to the veterans without PTSD (VETC), which was the case. There were no trauma‐specific effects and this slowing was to all threatening stimuli, regardless of category membership. This suggests a generalized threat‐processing interference rather than a trauma‐specific effect in these combat‐related PTSD patients (Pergamin‐Hight et al., 2015).

It is well‐established that individuals with PTSD allocate disproportionate attention to threatening or trauma‐related cues (Brewin, Kleiner, Vasterling, & Field, 2007; Ehlers, 2015), and some contend that this bias may underlie prolonged symptom expression, such as hyperarousal, in PTSD patients (Ehlers & Clark, 2000). Cognitive theories suggest that a heightened fear response primes threatening representations, subsequently facilitating activation of the fear network by trauma‐relevant cues (McNally, 2006). A large portion of evidence supporting this model comes from behavioral data gathered using experimental paradigms that range from lexico‐semantic color naming tasks (Cisler et al., 2011) to spatial attention tasks (Bryant & Harvey, 1997) to rapid target detection tasks (Amir, Taylor, Bomyea, & Badour, 2009). However, few of the aforementioned paradigms utilize visual stimuli that include both threatening trauma‐specific and threatening nontrauma specific stimuli. For example, Olatunji et al. (2013) implemented a rapid visual processing task and found trauma‐specific threat biases in combat‐related PTSD veterans, but combat‐related visual stimuli were the only threatening stimuli included. Although previous studies have found performance‐related threat interference for trauma‐specific stimuli, our findings indicate that when utilizing both threatening trauma‐related and threatening nontrauma related stimuli, behaviorally there is a general threat processing bias in PTSD patients.

Our results add additional support for cognitive models that posit facilitated or exaggerated activation of the threat network in individuals with PTSD (McNally, 2006). However, the mechanisms underlying this bias to threatening cues are not well‐understood. In attempts to disentangle the mechanisms contributing to this generalized threat interference, recent studies have merged behavioral, neurophysiological, and imaging techniques (Fani, Jovanovic, et al., 2012; Fani, Tone, et al., 2012). Fani, Tone, et al. (2012) measured physiological response during a fear‐startle paradigm and assessed threat bias using emotional faces. They found that participants with PTSD who exhibited a threat bias also showed an exaggerated startle response. When a similar task was implemented while acquiring functional neuroimaging, the threat bias found for the PTSD patients correlated with activity of the ventrolateral prefrontal cortex and the anterior cingulate (Fani, Jovanovic, et al., 2012). In our study, there was reduced fronto‐cortical theta activity, discussed below, suggesting a reduced cortical capacity to regulate the threat network (Cavanagh, Eisenberg, Guitart‐Masip, Huys, & Frank, 2013; Cavanagh & Shackman, 2015), consequently allowing the threatening cues to impede task related activity and thus to delay response time. This interpretation follows current neurocircuitry models of PTSD (Pitman et al., 2012); however, further study is needed to better understand the precise mechanisms involved in the general threat processing bias that we found for the veterans with PTSD.

### 4.2 Electrophysiology

Theta band activity is often found in memory related tasks and is associated with cortico‐limbic interactions in memory processes. In general, several studies using intracranial EEG and surface EEG show that theta activity plays a pivotal role in retrieving long‐term memories (Buzsáki & Draguhn, 2004; Fell & Axmacher, 2011). More specifically, studies have shown that theta power during memory retrieval is responsible for higher order memory control processes by means of coordinating activity of nonadjacent brain regions (Fries, 2009; Jensen & Colgin, 2007; Staudigl, Hanslmayr, & Bäuml, 2010; Womelsdorf et al., 2007). Various topographical patterns help guide interpretation, as theta power fluctuations over dispersed cortical regions vary according to the memory mechanisms involved. For example, frontal theta power changes are indicative of control processes in regards to monitoring and regulation of affective memories (Cavanagh et al., 2013; Cavanagh & Shackman, 2015), while, parietal and posterior theta power changes may relate to obtaining sensory information, as during a semantic word retrieval task (Bastiaansen, Oostenveld, Jensen, & Hagoort, 2008; Bastiaansen, Van Der Linden, Ter Keurs, Dijkstra, & Hagoort, 2005).

Previous investigations in healthy controls using these same stimuli and task demonstrated frontal theta to threatening stimuli (DeLaRosa et al., 2014). In this study, we found that frontal theta power for the PTSD group was lower than that for the VETC group for the threatening combat scenes, which may be indicative of disruption of cortico‐limbic communication (Brockmann, Pöschel, Cichon, & Hanganu‐Opatz, 2011; Buzsáki & Draguhn, 2004; Fujisawa & Buzsáki, 2011; Siapas, Lubenov, & Wilson, 2005). In healthy individuals, previous studies have found reduced frontal theta during a memory processing task following an acute stressor (Gärtner, Rohde‐Liebenau, Grimm, & Bajbouj, 2014). In addition, there are various neurochemical changes that occur following a stressful experience that impair prefrontal cortex functioning (Arnsten, 2009; McEwen & Morrison, 2013). Experiencing a stressful event involves activation of the sympathetic nervous system, increasing prefrontal catecholamine levels, and of the hypothalamus‐pituitary‐adrenal axis, increasing levels of prefrontal glucocorticoids (Diorio, Viau, & Meaney, 1993; Finlay, Zigmond, & Abercrombie, 1995; Roozendaal, Okuda, De Quervain, & McGaugh, 2006). Although this typical stress response is responsible for enhancing memory for emotional stimuli (McIntyre, Power, Roozendaal, & McGaugh, 2003), effects of prolonged activation of this system, as has been suggested may occur in PTSD, are less well understood. It is likely that long‐term effects of a hyperactive stress response leads to overall reduced prefrontal functioning, as high concentrations of prefrontal catecholamine levels and glucocorticoid levels impair prefrontal cortex functioning (Arnsten, Mathew, Ubriani, Taylor, & Li, 1999; Dominique, Roozendaal, & McGaugh, 1998; Gründemann, Schechinger, Rappold, & Schömig, 1998). The prefrontal reduction in theta power found in this study may be a result of prolonged activation of the stress response in the PTSD group resulting in reduced functioning of the prefrontal cortex.

This trauma‐specific frontal theta power effect showed a relationship with PTSD symptomatology. As predicted, we found that the PTSD group's frontal theta power for the threatening stimuli was lower than the VETC group's, which was driven by the trauma‐specific combat stimuli, the threatening OEF/OIF combat images. Frontal regions showed no group differences for the threatening animals, items, or nature scenes. In healthy individuals, it has been suggested that frontal cortical regions have a modulatory role on subcortical limbic regions, such as the amygdala, often inhibiting threat‐related behavior (Cavanagh et al., 2013; Cavanagh & Shackman, 2015; Courtin, Bienvenu, Einarsson, & Herry, 2013). In regard to negative emotional stimuli, the cingulate cortex in normal individuals has a regulatory role with respect to limbic emotional responses (Etkin, Egner, & Kalisch, 2011). Anatomical models of PTSD include structural and functional abnormalities of cingulate cortices and limbic regions (Etkin & Wager, 2007; Nutt & Malizia, 2004; Pitman et al., 2012). The most common findings regarding neurocircuitry in PTSD are a hyperresponsive amygdala and hyporeactive frontal cortices, resulting in failure of emotion modulation (Pitman et al., 2012). The trauma‐specific theta band results support such a model. It is likely that the trauma‐specific images involuntarily activate the fear‐processing network (Jovanovic & Norrholm, 2011), and the observed reduction in frontal theta for the threatening combat stimuli compared to the typical control response represents a lack of frontal inhibition of this response. The correlation analysis in this study may further support a modulatory role for frontal theta band activity. There was a negative correlation between differential frontal theta power to the combat stimuli and the hyperarousal subscore of the CAPS (Blake et al., 1990). For the PTSD group, a high hyperarousal CAPS score was associated with a low frontal theta band power to the trauma‐specific combat stimuli. Further characterization of frontal theta and its relationship with PTSD symptomatology may inform future therapeutic interventions.

Clinical research consistently reports a relationship between mood disorders and a negativity bias (Bryant & Harvey, 1997; Fani, Jovanovic, et al., 2012; van den Heuvel et al., 2005). Emotion negativity bias plays a role of maintenance in anxiety, with changes in automatic threat processing preceding and predicting clinical changes (Reinecke, Rinck, Becker, & Hoyer, 2013; Reinecke, Waldenmaier, Cooper, & Harmer, 2013). Due to the difficulty in treating mood disorders, clinical interest in alternative treatments, such as neuromodulation techniques, has increased. For example, transcranial direct current stimulation (tDCS) of the dorsolateral prefrontal cortex (DLPFC) has been shown to reduce vigilance to threatening stimuli (Ironside, O'Shea, Cowen, & Harmer, 2016), and increasing activity in the DLPFC with tDCS causally contributes to the modification of negativity bias (Clarke, Browning, Hammond, Notebaert, & MacLeod, 2014). Meta‐analyses also have shown efficacy for PTSD symptom reduction with lower (e.g., 1 Hz) and higher (e.g., 10 Hz) frequencies (Berlim & Van den Eynde, 2014; Trevizol et al., 2016), and more recent research has potential efficacy of theta‐burst rTMS stimulation (Philip et al., 2019). Our findings showing differences in theta power with regard to threatening stimuli may serve as potential therapeutic targets for neuromodulation interventions.

### 4.3 Limitations

It is important to note the limitations of our study. First, combat exposed veteran of OEF/OIF served as the control group; however, the effects of traumatic combat exposure are not currently well understood (Bunce, Larson, & Peterson, 1995; Prigerson, Maciejewski, & Rosenheck, 2002). It would be informative to have a second control group of healthy nontrauma exposed individuals to further investigate the role of frontal theta in response to threatening stimuli. Also, while interpreting our findings it is important to have caution regarding premorbid neurobiological conditions versus changes caused by a traumatic event (Kremen, Koenen, Afari, & Lyons, 2012). In addition, statistical power is low due to the small sample size.

## 5 CONCLUSION

We found trauma‐specific effects in frontal regions of theta band EEG activity in veterans of OEF/OIF with PTSD. For the PTSD group, there was reduced cortical capacity to exert cognitive control, as measured by reduced frontal theta power for the threatening trauma‐specific images, and this reduction in theta power negatively correlated with hyperarousal symptoms. Behavioral data revealed a more general threat processing bias, as measured by slower reaction times to threatening stimuli for the PTSD group than for the VETC group, suggesting that the threatening images impeded task relevant responses. These findings complement and extend previous studies of PTSD that implicate a hyperactive limbic system with a reduced cortical capacity to exert inhibition.

## CONFLICT OF INTEREST

The authors report no financial or potential conflicts of interest.

## Data Availability

Not Applicable.
